# Contemporary habitat discontinuity and historic glacial ice drive genetic divergence in Chilean kelp

**DOI:** 10.1186/1471-2148-10-203

**Published:** 2010-07-01

**Authors:** Ceridwen I Fraser, Martin Thiel, Hamish G Spencer, Jonathan M Waters

**Affiliations:** 1 Allan Wilson Centre for Molecular Ecology and Evolution, Department of Zoology, University of Otago, 340 Great King St, Dunedin 9016, New Zealand; 2 Facultad Ciencias del Mar, Universidad Católica del Norte, Larrondo 1281, Coquimbo, Chile; 3 Centro de Estudios Avancados en Zonas Aridas (CEAZA), Coquimbo, Chile

## Abstract

**Background:**

South America's western coastline, extending in a near-straight line across some 35 latitudinal degrees, presents an elegant setting for assessing both contemporary and historic influences on cladogenesis in the marine environment. Southern bull-kelp (*Durvillaea antarctica*) has a broad distribution along much of the Chilean coast. This species represents an ideal model taxon for studies of coastal marine connectivity and of palaeoclimatic effects, as it grows only on exposed rocky coasts and is absent from beaches and ice-affected shores. We expected that, along the central Chilean coast, *D. antarctica *would show considerable phylogeographic structure as a consequence of the isolating effects of distance and habitat discontinuities. In contrast, we hypothesised that further south - throughout the region affected by the Patagonian Ice Sheet at the Last Glacial Maximum (LGM) - *D. antarctica *would show relatively little genetic structure, reflecting postglacial recolonisation.

**Results:**

Mitochondrial (COI) and chloroplast (*rbc*L) DNA analyses of *D. antarctica *from 24 Chilean localities (164 individuals) revealed two deeply divergent (4.5 - 6.1% for COI, 1.4% for *rbc*L) clades from the centre and south of the country, with contrasting levels and patterns of genetic structure. Among populations from central Chile (32° - 44°S), substantial phylogeographic structure was evident across small spatial scales, and a significant isolation-by-distance effect was observed. Genetic disjunctions in this region appear to correspond to the presence of long beaches. In contrast to the genetic structure found among central Chilean populations, samples from the southern Chilean Patagonian region (49° - 56°S) were genetically homogeneous and identical to a haplotype recently found throughout the subantarctic region.

**Conclusions:**

Southern (Patagonian) Chile has been recolonised by *D. antarctica *relatively recently, probably since the LGM. The inferred trans-oceanic ancestry of these Patagonian populations supports the notion that *D. antarctica *is capable of long-distance dispersal via rafting. In contrast, further north in central Chile, the correspondence of genetic disjunctions in *D. antarctica *with long beaches indicates that habitat discontinuity drives genetic isolation among established kelp populations. We conclude that rafting facilitates colonisation of unoccupied shores, but has limited potential to enhance gene-flow among established populations. Broadly, this study demonstrates that some taxa may be considered to have either high or low dispersal potential across different temporal and geographic scales.

## Background

Oceanic ecosystems have historically been considered relatively open, with high connectivity among populations [1]. Recent studies have, however, demonstrated considerable population structure in a variety of coastal taxa (see [2-5]; and references therein), indicating that connectivity among marine populations is often lower than previously assumed [4]. Levels of connectivity in coastal ecosystems may be affected by a broad range of factors, including oceanography, geological history, habitat continuity, ecology and life history [6]. The relative importance of these factors in structuring marine populations, however, remains largely unknown [7].

Genetic patterns of isolation-by-distance are common in the marine realm, with neighbouring populations generally more closely related to each other than to those further away, as the chance of successful dispersal decreases with increasing distance [3,6]. Gene flow among populations with particularly stringent ecological requirements may also be impeded by habitat discontinuities [2,8]. Extensive stretches of sandy beach might, for example, represent important biogeographic hurdles for rocky-shore marine taxa, even those with a planktonic larval stage [7,9-12]. The genetic effects of habitat discontinuities should be greatest among populations of taxa with relatively low dispersal potential. Indeed, in several species of marine macroalgae with predicted low dispersal potential, discontinuities in suitable substratum have been linked to reduced gene flow among populations [13-15].

Although most macroalgae have only short-lived gametes or dispersive zygotes, some - particularly highly buoyant species - are hypothesised to be capable of long-distance dispersal via rafting of fertile adults (e.g., *Fucus vesiculosus*: [16]; *Hormosira banksii*: [17]; *Macrocystis pyrifera*: [18]). A broad-scale phylogeographic study of one such species, southern bull-kelp (*Durvillaea antarctica*), found evidence of recent (postglacial) recolonisation of much of the subantarctic, a feat almost certainly achieved by trans-oceanic rafting of adult specimens [19]. Recolonisation of the subantarctic was inferred from extremely low levels of genetic structure among bull-kelp populations throughout this region, versus high levels of genetic structure in glacial refugia (e.g., New Zealand). Low levels of genetic structure of populations in historically glaciated regions, versus relatively high structure in unglaciated regions, is a signature of postglacial recolonisation that has been demonstrated in a wide range of marine and terrestrial taxa (reviewed by [20-24]). But if bull-kelp is able to disperse over long distances by rafting, how is high genetic structure maintained in refugial regions? Fraser et al. [19] suggested that the genetic structure observed in refugial areas may be largely a consequence of the density-blocking effects of established populations [20,25]. In dense kelp populations, any limited available substratum is more likely to be colonised by zygotes from residents rather than those from rare, rafted individuals. Phylogeographic structure within refugial areas may therefore be driven more through isolation-by-distance, or breaks in habitat continuity, than by rafting dispersal. Rafting may, in contrast, enable long-distance dispersal and colonisation of unoccupied habitats.

**Figure 1:**
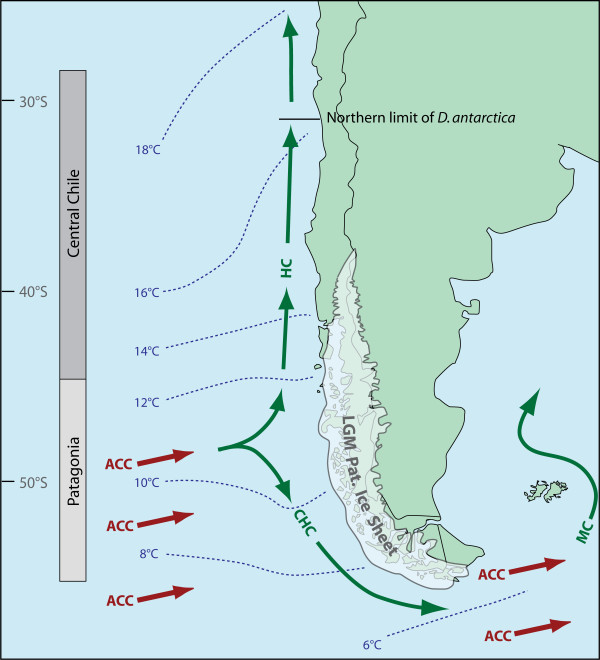
**Patagonian Ice Sheet at the Last Glacial Maximum**. Map of South America showing the extent of the Patagonian Ice Sheet at the LGM (after [27]). Modern oceanographic conditions around South America are also indicated. Dashed lines indicate mean annual sea surface temperatures (after [80]). Arrows show directions of major surface currents, the Antarctic Circumpolar Current (ACC), the Humboldt Current (HC), the Cape Horn Current (CHC) and the Malvinas Current (MC) (after [81]).

Chile presents an intriguing system for assessing the relative phylogeographic impacts of isolation-by-distance, habitat discontinuities, and historic glaciations. Central Chile has an almost straight coastline characterised by numerous extensive stretches of beach that isolate rocky-shore habitats [8]; this coast was largely unaffected by ice during the Pleistocene glaciations [26]. Further south, Chilean Patagonian shores also form isolated habitats of islands and peninsulas, but at the Last Glacial Maximum (LGM), an extensive ice sheet covered most of this region [27,28] (Fig. 1). This glacial ice probably extended westward to the edge of the continental shelf [28], and likely extirpated many populations of rocky shore taxa. Such contrasts in geological history between central and Patagonian Chile should be reflected in the phylogeographic structure of taxa occurring across both regions. *Durvillaea antarctica *currently dominates the rocky shores of Chile from Cape Horn [29], at around 56°S, up to approximately 32°S [8]. Fraser et al.'s [19] recent broadscale study of *D. antarctica *included few samples from central Chile (only seven localities), and none from Chilean Patagonia, and was therefore unable to assess phylogeographic contrasts along the Chilean coast. For the current study, we analysed samples from a total of 24 localities, encompassing the full range of *D. antarctica *in Chile, to test the predictions that (i) *D. antarctica *populations in central Chile would show strong phylogeographic structure, and that (ii) *D. antarctica *populations in southern Chile would show little genetic structure, reflecting postglacial recolonisation.

## Results

DNA sequencing of 164 *D. antarctica *samples from 24 localities along the coast of Chile yielded 14 distinct haplotypes for mitochondrial COI, with 34 variable sites detected across the 629 bp fragment. For a chloroplast marker, *rbc*L, 78 sequences from nine localities (six in central Chile: Table 1) yielded two haplotypes, with 12 variable sites across the 886 bp fragment. Twenty-seven of the 34 variable COI nucleotide sites were third-codon positions, and transitions accounted for most inferred changes, with transversions inferred at only three sites for COI, and at one site for *rbc*L. As no genetic diversity for *rbc*L was observed in 54 samples from six widely-spaced central Chilean localities, obtaining *rbc*L sequences for all samples for this region was deemed redundant, and only a subset of samples were analysed for this less-informative marker.

**Table 1:** Sample localities and number of samples sequenced from each for both mitochondrial (COI) and chloroplast (*rbc*L) DNA from *D. antarctica*.

Locality name	Latitude	Longitude	#samples sequenced COI	#samples sequenced *rbc*L
Pichicuy*	32° 20' 43.92S	71° 27' 36.07W	10	8

Montemar*	32° 57' 27.31S	71° 33' 03.67W	10	10

Cartagena	33° 33' 3.06S	71° 37' 0.11W	6	

Matanzas	33° 45' 57.27S	71° 46' 14.68W	5	

Pichilemu	34° 23' 37.79S	72° 01' 34.10W	6	

Duao	34° 53' 12.37S	72° 10'' 07.14W	7	

Constitución*	35° 19' 41.18S	72° 25' 59.41W	6	9

Dichato	36° 29' 41.88S	72° 54' 36.26W	6	

Tumbes*	36° 36' 55.44S	73° 06' 29.74W	8	9

Lebu	37° 35' 42.00S	73° 40' 9.00W	5	

Tirua	38° 20' 42.57S	73° 30' 30.27W	4	

Queule	39° 23' 16.66S	73° 14' 26.60W	5	

Punta Loncoyen*	39° 49' 28.09S	73° 24' 18.68W	9	9

Bahia Mansa*	40° 35' 34.21S	73° 45' 01.30W	5	

Pumillahue	41° 56' 44.99S	74° 02' 22.02W	8	

Cucao*	42° 40' 8.04S	74° 07' 18.55W	10	9

Isla Guafo	43° 33' 17.22S	74° 42' 55.69W	5	

Puerto Barrientos	43° 54' 44.39S	74° 0' '7.31W	6	

Isla Betecoi	43° 59' '7.62S	73° 52' 23.30W	9	

49°S	49° 09' 24.94S	75° 17' 48.99W	7	10

50°S	50°08' 56.76S	74°39' 52.56W	10	10

51°S	51°46' 43.7S	73°43' 00.6W	6	

53°S	53° 39' 19.13S	72° 15' 29.02W	7	

Cape Horn	55° 59' 31.47S	67° 16' 14.98W	4	4

**TOTAL Chile**			**164**	**78**

* Asterisks indicate localities included in previously-published analyses [19].

**Figure 2:**
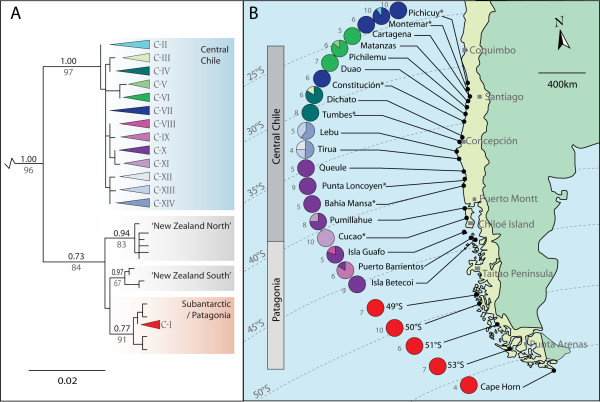
**Phylogeography of Chilean bull-kelp**. A) ML phylogeny of *D. antarctica *for COI including data from 17 new localities in Chile, as well as published sequences from 7 localities in central Chile (indicated by asterisks), and localities in New Zealand and the subantarctic [19]. Coloured triangles represent haplotypes found in Chile, with colours corresponding to those in panel B and Fig 3. Bayesian PP values are shown in black above the line, and ML bootstraps are in grey below the line. Support values < 50% are not shown, nor are values on some minor, distal branches within the major clades. Outgroups have been trimmed for clarity. B) Location of all sampled localities along the coast of Chile. Pie charts indicate distribution and proportions of haplotypes, with haplotype colours corresponding to those in panel A and Fig 3. Grey numbers to the left of pie charts show total number of samples from each locality.

**Figure 3:**
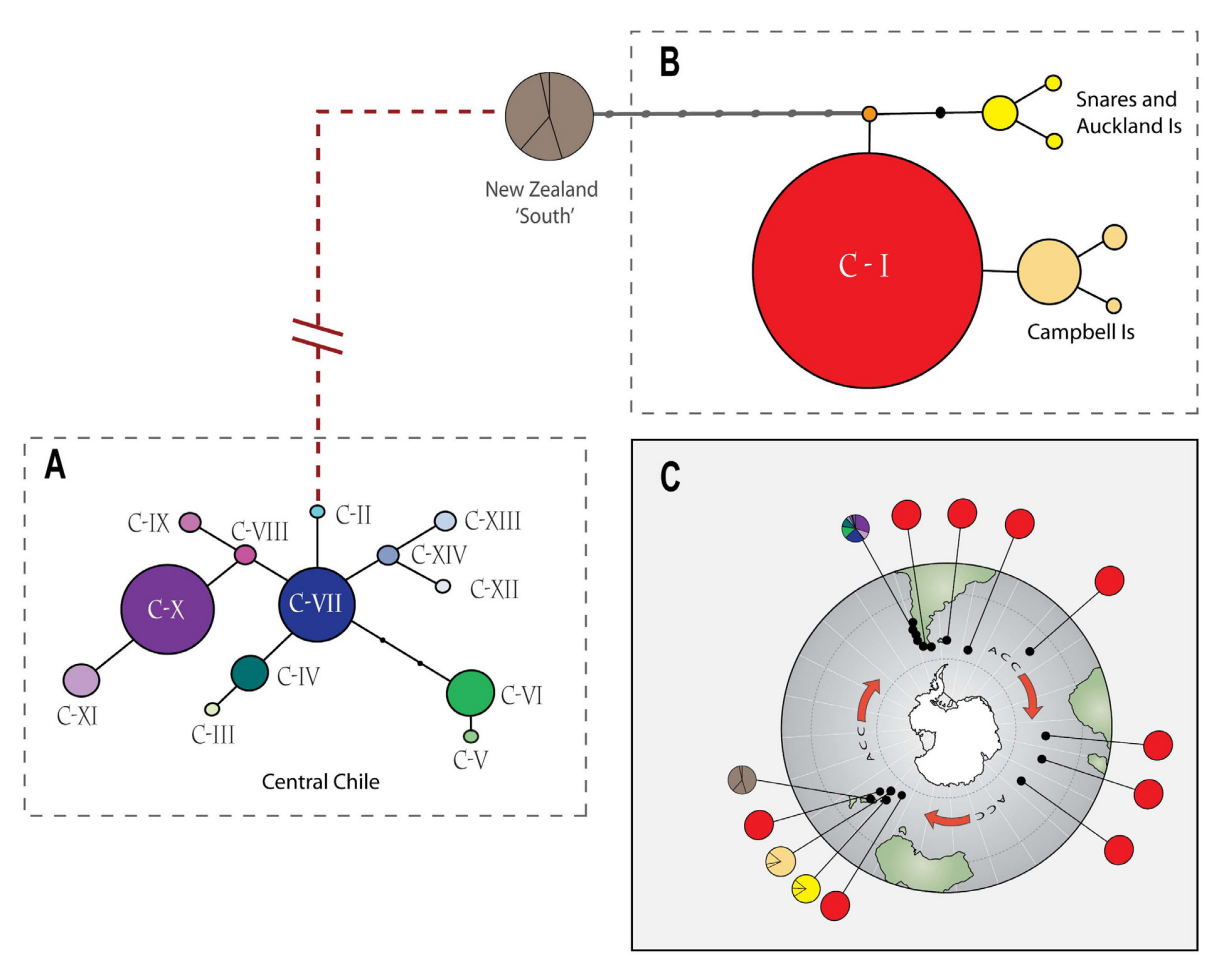
**Haplotype networks and global projection**. COI haplotype network diagrams of *D. antarctica *for A) the 'central Chilean' clade and B) the 'Patagonian/subantarctic' clade (see also [19]), with circle size scaled approximately according to haplotype frequency, and small dots representing undetected, hypothetical haplotypes. Although the 'Patagonian/subantarctic' and 'New Zealand south' clades [19] joined parsimoniously using network analysis, the 'central Chilean' clade did not join to any other lineages at ≥90% confidence limit: a hypothetical, non-statistically supported connection between the major clades is indicated with a red dashed line. The most common (C-I/red) haplotype was found throughout Chilean Patagonia [this study] and the subantarctic [19]. C) Global projection showing locations of other (subantarctic) sites at which the Patagonian (C-I/red) haplotype has previously been detected [19]. The eastward flow of the Antarctic Circumpolar Current (ACC) is indicated.

Phylogenetic analyses of COI revealed that 13 of the *D. antarctica *haplotypes in Chile (C-II to C-XIV) were restricted to populations in central Chile, whereas all sampled Chilean Patagonian populations south of 44°S were fixed for a single haplotype (C-I), which was highly divergent from the others (Fig. 2). The widespread Patagonian haplotype, C-I, was previously detected at numerous subantarctic locations throughout the Southern Hemisphere [19] (Fig. 3). With the exception of the Patagonian haplotype (C-I), all (central) Chilean haplotypes (C-II - C-XIV) were closely related to one another (uncorrected distances 0.2 - 1.3%), together forming a strongly supported clade (Bayesian PP 1.00; Fig. 2a); by contrast, the Patagonian haplotype (C-I) was markedly divergent from all other Chilean haplotypes (C-II - C-XIV) (uncorrected distances 4.5 - 6.1%). The two chloroplast (*rbc*L) haplotypes detected in Chile were 1.4% divergent (uncorrected distance), with one (R-I) restricted to all sampled Patagonian populations, and the other (R-II) found only in central Chile (Cucao, Punta Loncoyen, Tumbes, Constitución, Montemar and Pichicuy). More broadly, the Patagonian *rbc*L haplotype R-I has a circum-subantarctic distribution, including the Falkland Islands and South Georgia (not shown; [19]).

Network analyses of Chilean COI haplotypes recovered two parsimonious networks that could not be joined at ≥90% confidence limits, with one comprising only the Patagonian haplotype C-I. All other Chilean haplotypes (CII-CXIV) formed a single network (Fig. 3 shows these networks in the context of previously-published work [19]).

**Figure 4:**
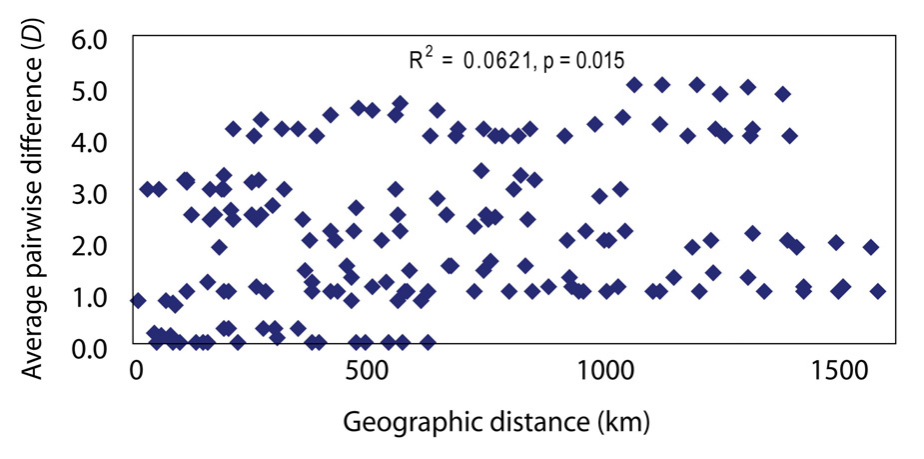
**Isolation-by-distance**. Isolation-by-distance (Mantel) analysis, illustrating the relationship between COI genetic distance (Nei's raw average pairwise difference, *D*) and geographical distance for *D. antarctica *among central Chilean (32°-42°S) sampling locations.

**Figure 5:**
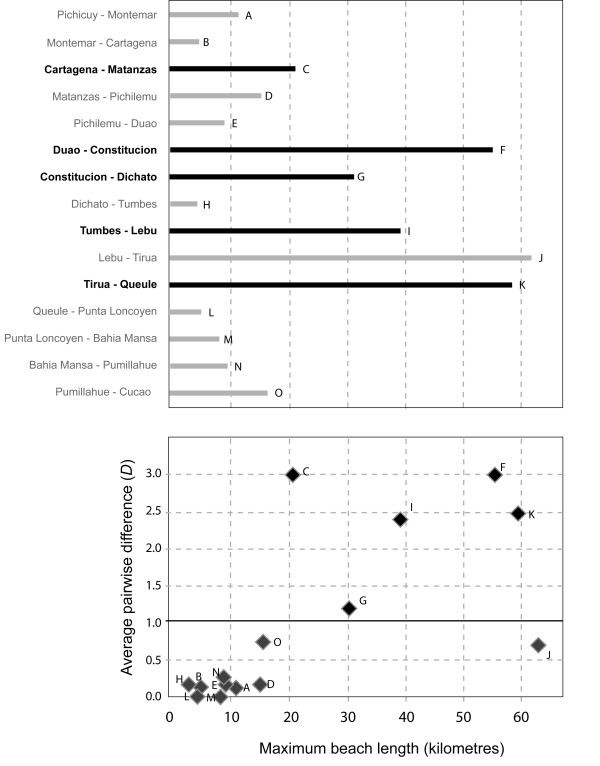
**The effect of beach length on phylogeographic structure in bull-kelp in central Chile**. Maximum length of beaches between pairs of adjacent sampled localities in central Chile [data estimated from Google Earth satellite images]. Upper: locality pairs between which there is a marked mitochondrial (COI) genetic disjunction (Nei's raw average pairwise difference, D, > 1.0) are shown in black, whereas genetically-similar locality pairs (D < 1.0) are shown in grey. Lower: Nei's raw average pairwise differences (D) for COI, plotted against maximum uninterrupted beach length, between all adjacent locality pairs in central Chile.

The Mantel test revealed a significant relationship between genetic and geographic distance (*P *= 0.015 for 999 permutations) (Fig. 4). Detailed geographic analyses based on maximum uninterrupted beach length between pairs of adjacent sampling locations in central Chile revealed a clear relationship between rocky shore habitat continuity and genetic connectivity along the central Chilean coast (Fig. 5). Under the definition of genetic disjunction corresponding to Nei's *D *≥1.0, no 'disjunct' locality pairs shared any haplotypes. Notably, all phylogeographic disjunctions in central Chilean *D. antarctica *were associated with extensive (> 20 km) beaches (Fig. 5). The only adjacent locality pair separated by a beach > 20 km long that did *not *show a genetic disjunction was Lebu/Tirua (locality pair J in Fig. 5), separated by 62 km of uninterrupted beach. Logistic regression (with the binomial response variable of 'genetically disjunct or not', and the continuous fixed effects 'maximum beach length between adjacent localities' and 'coastal distance between adjacent localities') revealed that maximum beach length was a significant predictor of genetic disjunction (β = 0.05; *P *= 0.035; *N *= 15), whereas total coastal distance was not (β = 0.01; *P *= 0.608; *N *= 15). The outlier formed by Lebu/Tirua (locality pair J, Fig. 5) is, however, likely having a strong effect on the analysis; indeed, removing this locality pair from the analyses greatly strengthens the effect of long beaches in the logistic regression (β = 0.23; *P *= 0.002; *N *= 14). With or without the outlier, these results indicate that habitat discontinuity influences genetic connectivity among populations of central Chilean bull-kelp.

## Discussion

Two broad findings were revealed by our phylogeographic research of *D. antarctica *in Chile. First, substantial phylogeographic structure was observed in central Chile (hypothesis i). Genetic isolation among populations in this region appears driven by habitat discontinuities. Second, and in direct contrast to the pattern observed for central Chile, samples from Chilean Patagonia showed complete genetic homogeneity within and among localities (hypothesis ii). These Patagonian populations were deeply divergent to those from more northern (central Chilean) localities, and appear to have trans-oceanic ancestry.

### Habitat discontinuity and population connectivity in central Chile

The substantial phylogeographic structure observed among central Chilean sampling localities of *D. antarctica *(Fig. 2) suggests a low level of genetic connectivity across this region (hypothesis i), a finding supported by the significant results of the isolation-by-distance test. Furthermore, the strong relationship between long beaches and genetic disjunctions in *D. antarctica *(Fig. 5) suggests that habitat discontinuity is likely a major factor controlling local genetic connectivity in this rocky-shore macroalga. Population connectivity of other kelp species may be similarly affected by interruptions in habitat: Faugeron et al. [15], for example, demonstrated genetic differentiation among populations of the kelp *Lessonia nigrescens *in northern Chile across a habitat discontinuity formed by a mine-waste disposal site. In the English Channel, Billot et al. [13] found evidence for reduced gene flow among populations of the kelp *Laminaria digitata *across habitat discontinuities. Patterns of genetic disjunction associated with habitat discontinuity have also been noted for numerous rock-dwelling taxa with predicted high dispersal abilities, including rocky-reef fish [7,11], rock-pool copepods [9], and macroalgae (e.g., small-scale patterns of genetic structure in *Phyllospora *[12]), suggesting that for marine taxa, as observed for terrestrial plants [30,31], dispersive life history alone does not necessarily lead to panmixia.

That habitat discontinuity appears to disrupt connectivity among central Chilean populations of *D. antarctica *is in direct contrast to the broad evidence of long-distance, trans-oceanic dispersal of this species in the subantarctic [19]. Several biological factors may help to explain such a contrast in apparent dispersal ability/effectiveness. First, established and dense kelp populations may resist genetic input from rafted individuals [19] in a form of density-blocking [20,25]. In such populations, limited substratum availability and high competition from local *D. antarctica *zygotes would suppress settlement of zygotes from such migrants. Conversely, shores swept clean by ice [19] or other large-scale disturbances would offer ample settlement opportunities for zygotes from rafted migrants. Although the genetic effects of density-blocking have yet to be demonstrated experimentally for brown algae, the complete genetic homogeneity of putative postglacially-recolonised populations (this study and [19]), and the absence of the Patagonian COI haplotype (CI) from central Chilean populations, indicate that such a process is likely to be occurring. Second, although *D. antarctica *is clearly an effective long-distance disperser, there may be differences in reproductive viability of rafts across latitudinal gradients. Numerous reports suggest that *D. antarctica *has an extended period of fertility in the subantarctic versus mainland New Zealand or Chile (see references in [29]), perhaps facilitated by longer optimal reproductive conditions in colder waters. Observations by Macaya et al. [18] suggest that detached rafts of another kelp, *Macrocystis pyrifera*, lose reproductive activity at high temperatures, a finding supported by Rothäusler et al. [32]. If *D. antarctica *rafts are similarly affected by high water temperatures, fewer zygotes from immigrant individuals may be available to colonise central Chile than in the relatively cold waters of Chilean Patagonia (Fig. 1).

Local genetic structure within and among central Chilean *D. antarctica *populations could additionally be influenced by a range of processes such as seasonal variation and patchy recruitment [33] or harvesting [34-36]. While the results from Bustamante & Castilla [35] and Castilla et al. [36] underline the importance of short-distance dispersal in local population dynamics, coastal islands might serve as stepping stones allowing dispersal across long stretches of sandy beaches. Indeed, the only locality pair separated by a long beach that did *not *show a genetic disjunction (Lebu-Tirua) is in the immediate vicinity of a large coastal island (Isla Mocha) that may effectively connect the two populations despite the beach barrier. Interestingly, Constitución, flanked by long beaches on both sides, was genetically distinct from adjacent localities but not from some more distant localities (Cartagena, Montemar and Pichicuy). These populations, on the northernmost edge of the range of *D. antarctica *in Chile, may be sensitive to any changes in water temperature. Changes to the northern range limit of an Australian congeneric, *D. potatorum*, have been linked to warming waters in recent years [37]. The northern populations of *D. antarctica *in Chile (Cartagena, Montemar and Pichicuy) may thus be somewhat ephemeral in the long term, and could have been relatively recently colonised by kelp drifting from southern populations (e.g., Constitución) in the north-flowing Humboldt Current.

### Major genetic disjunction in southern Chile

Our phylogeographic analyses of Chilean *D. antarctica *populations indicate a biogeographic break in northern Chilean Patagonia (44° - 49°S), with both mitochondrial and chloroplast DNA phylogenies revealing a deep genetic disjunction between samples from southern Patagonia and those from central Chile (Fig. 2). Although the precise geographic location of this major genetic disjunction cannot be pinpointed with this present dataset, a biogeographic break in the vicinity of the Golfo de Penas/Taitao Peninsula (46.5°S; Fig. 2) has previously been noted [38,39]. More recently, Lancellotti and Vásquez [40] suggested this feature may represent the southern boundary of the transitional zone, separating it from the cold-temperate southern waters south of 46°S. The hypothesis of a biogeographic break at Taitao Peninsula has been supported by several recent shallow-marine surveys of Cnidaria in southern Chile [41,42], with samples showing distinct changes in species composition to the north and south of about 46°S. In a recent study of the density of drifting kelp in channels north of Taitao Peninsula, Hinojosa et al. [43] suggest that *D. antarctica *rafts are driven by both surface currents (primarily westward) and wind (primarily eastward) through the fiords. Taitao Peninsula, however, projects across the dense system of channels that could otherwise potentially allow mixing and north-south transport of drifting *D. antarctica *within the fiords.

The deep genetic disjunction between *D. antarctica *samples from southern Patagonia and those from central Chile raises the question of the taxonomic status of these two divergent lineages. A recent study of *D. antarctica *revealed that this taxon comprises two ecologically, morphologically and genetically distinct forms in New Zealand [44]. Given that the genetic disjunction between Patagonian and central Chilean *D. antarctica *(4.5 - 6.1% for COI) exceeds the divergence observed between the co-occurring, genetically-distinct forms in New Zealand (3.0 - 3.8% for COI: [44]), it is possible that the distinct Chilean lineages represent reproductively-isolated species, and future studies should assess any physiological, ecological or life history differences between them. Regardless, the phylogeographic contrasts between these lineages are intriguing, with negligible genetic structure observed throughout sampled Patagonian populations (a single haplotype for COI at all five localities across some 1000 km of shoreline), versus high structure further north (eleven COI haplotypes across approximately 1400 km).

### Postglacial recolonisation of coastal Chilean Patagonia

The complete genetic homogeneity observed in *D. antarctica *from all localities south of latitude 44°S (the region covered by the Patagonian Ice Sheet at the LGM: Fig. 1), compared with substantial phylogeographic structure among populations in central Chile for COI (Fig. 2), strongly supports a hypothesis of postglacial recolonisation of Chilean Patagonia (hypothesis ii). Such events are typically marked by low levels of genetic structure in recolonised versus refugial areas (reviewed by [20-24]) although, to date, few studies have been carried out in the Southern Hemisphere [45]. Recolonisation of Patagonia appears to have occurred from a trans-oceanic source, rather than from geographically proximate areas in central Chile. The Patagonian COI haplotype C-I also occurs on numerous subantarctic islands (Fig. 3, and see [19]), and is more closely related to haplotypes found in the New Zealand subantarctic region than to other Chilean haplotypes [19], suggesting the New Zealand region as a source, with transport facilitated by the Antarctic Circumpolar Current (Fig. 1) [46-49].

Although genetic evidence of postglacial recolonisation has previously been identified from a variety of Patagonian terrestrial and freshwater taxa, including fish (galaxiids: [50-52]; percichthyids: [53,54]), lizards [55], and plants [56-58], this is the first study to reveal evidence of postglacial recolonisation of a Chilean Patagonian marine taxon (but see recent findings on the likely impacts of glacial cycles on marine dispersal by diadromous fish in Patagonia [51]). Additionally, in comparison with Fraser et al. [19], who inferred recolonisation of several subantarctic islands by *Durvillaea *after extirpation by sea ice, this work shows that the glacial (i.e., land-based) ice during the most recent ice age may also have driven kelp population extinctions. If populations of *D. antarctica *in southern Chile were indeed destroyed by the Patagonian Ice Sheet during the last glacial period, numerous other coastal taxa may have been similarly affected. Intriguingly, a broad-scale phylogeographic study of the rocky-shore gastropod *Concholepas concholepas *[59], found no evidence of recent (post-LGM) recolonisation of Chilean Patagonia. Perhaps the habitat of this molluscan species, extending to 40 m below sea level [60], enabled it to survive throughout the last ice age in regions where more intertidally-restricted taxa were eliminated by ice. Future studies should aim to assess whether a pattern of postglacial recolonisation is common among other subtidal and intertidal species in southern Chile.

## Conclusions

Our understanding of evolutionary processes relies largely on our knowledge of the factors that drive and maintain genetic variation and population connectivity. Marine systems can present paradoxical patterns of genetic connectivity, with taxa that apparently have low intrinsic dispersal capacities occasionally showing broader geographic ranges than highly dispersive taxa [61-63]. In such cases, passive dispersal by rafting may explain the broad distributions of otherwise non-dispersive species [64]. The results of the current study suggest that some taxa cannot necessarily be classified as either 'high' or 'low' dispersal organisms, and that mechanisms of dispersal may vary across temporal and geographic scales. Bull-kelp (*D. antarctica*) is capable of long-distance dispersal via rafting of fertile adults, as evidenced by the trans-oceanic ancestry of populations throughout postglacially-recolonised Chilean Patagonia (and subantarctic islands [19]). Intriguingly, this dispersal capacity facilitates colonisation of as-yet unoccupied rocky shores, but does not enhance connectivity among established populations. Long-established populations, such as those along the coast of central Chile, seem strongly influenced by the isolating effects of geographic distance and habitat discontinuities, indicating relatively poor inter-population connectivity.

## Methods

### Sample collection, DNA extraction and sequencing

164 samples of *D. antarctica *were collected from 24 localities along the coast of Chile, from Pichicuy (32°27'37"S) to Cape Horn (55°59'31"S) (Fig. 2, Table 1). These localities encompassed most of the Chilean range of this species, which extends from Los Vilos (31°54'27"S; 60 km north of Pichicuy) to Cape Horn. The data from seven of the localities in Chile (Cucao, Bahia Mansa, Punta Loncoyen, Tumbes, Constitución, Montemar and Pichicuy) were included in previously published analyses [19] but are here reanalyzed in-depth in the light of extensive additional sampling (106 new samples from 17 new localities). Samples consisted of small (< 2 cm^2^) pieces of tissue cut from the tip of healthy fronds of attached *D. antarctica*, and were preserved by desiccation in either 96% ethanol or silica gel beads. DNA extraction, amplification, purification and sequencing were performed as described in Fraser et al. [44]. A 629 bp region of mitochondrial cytochrome *c *oxidase I (COI) was amplified using primers GazF1 and GazR1 [65] for all samples. In addition, an 886 bp region of chloroplast *rbc*L was amplified using primers KL2 and KL8 [66] for samples from three localities in Chilean Patagonia, and analysed with previously-published sequences from central Chile (Pichicuy, Montemar, Constitución, Tumbes, Punta Loncoyen and Cucao) (Table 1).

### Analyses

Phylogenies for each DNA dataset were constructed using maximum-likelihood (ML) and Bayesian analyses, and included outgroup sequences from *Durvillaea willana *(South Island, New Zealand; GenBank accessions: EU918569 for COI and EU918578 for *rbc*L), *Durvillaea potatorum *(Tathra, southeastern Australia; GenBank accessions: FJ873092 for COI and FJ872990 for *rbc*L) and *Fucus *(GenBank accessions: *F. vesiculosus *AY494079 for COI and *F. gardneri *AF195515 for *rbc*L). To place the new Chilean sequence data in a wider context, published *D. antarctica *DNA sequences (GenBank accessions FJ550086 - FJ550112[19]) from seven of the localities in central Chile, and numerous localities around the subantarctic and New Zealand, were included in the analyses (Fig. 2). Note that the unique COI and *rbc*L haplotypes reported from the Crozet Islands by Fraser et al. [19] were later found to be erroneous, and were in fact identical to the widespread haplotypes C-I and R-I; this error was corrected for the present analyses. ML analyses were performed using PhyML [67] with a TrN + I [68] model for both COI and *rbc*L (proportion of invariable sites estimated, Nst = 6) as selected by the AIC of Modeltest 3.06 [69]. Node support was estimated by bootstrapping [70], with heuristic analysis of 1000 replicate data sets (Fig. 2). Bayesian posterior probability (PP) values were calculated using MRBAYES 3.1.2 [71] and are shown on the ML phylogenies (Fig. 2). Markov chain Monte Carlo (McMC) searches were performed, with four chains of 5,000,000 generations and trees sampled every 100. The first 10,000 trees sampled were discarded as 'burn-in,' as determined using Tracer v1.4 [72], while a consensus topology and posterior probability values were calculated with the remaining trees. Unrooted statistical parsimony networks were built using TCS 1.21 [73]. All unique DNA sequences obtained during this study were deposited with GenBank (accession numbers for all Chilean *D. antarctica *COI and *rbc*L sequences used in this study: FJ550093-FJ550095; FJ550097; FJ550099; FJ550119; HM103936-HM104173).

Analysis of isolation-by-distance was performed on data from the 19 localities in central Chile (north of 44°S) via the Mantel [74] test, implemented in GenAlEx6 [75], using raw (*D*) average pairwise differences [76] (calculated using Arlequin version 3.0 [77]) versus coastal distance (not including deep bays with narrow, < approximately 2 km, openings) among sample localities. Linearised population pairwise *F*_ST _values could not be used in the Mantel test: several localities were genetically monomorphic for different haplotypes, and pairwise comparisons between such fixed populations gave an *F*_ST _of 1.0; hence for these locality pairs no linearised *F*_ST _values (*F*_ST_/(1 - *F*_ST_), [78]) could be calculated.

Approximate measurements of the lengths of all beaches (coastal stretches of uninterrupted sand, gravel or pebbles) between pairs of adjacent rocky coastal localities in central Chile were made using the 'path ruler' tool in Google Earth http://earth.google.com/ to assess the continuity of suitable rocky shore habitat for *D. antarctica *among localities. As Chilean shores south of about 43°S are almost completely rocky, with few sandy beaches and, moreover, many wide oceanic channels between islands and peninsulas, measurements of beaches were only made for continental Chile and the coast of the Great Island of Chiloé (north of 43°S). The following analysis was performed only on data from sampled localities in this region (Pichichuy, at 32° 20', to Cucao, at 42°40' S). The isolating effects of beach length and total coastal distance between localities in central Chile were assessed by logistic regression. Adjacent locality pairs were first classified binomially as either genetically disjunct (*D *≥1.0) or not (*D *< 1.0). Under this definition, locality pairs not sharing any haplotypes were considered genetically disjunct, whereas those with shared haplotypes were not considered disjunct. Logistic regression was then carried out using R [79], using the logistf add-on package, with the model: 'genetically disjunct' ~ 'maximum beach length between adjacent localities' + 'coastal distance between adjacent localities'. This analysis enabled assessment of the relative abilities of total coastal distance, versus beach length, to predict whether or not adjacent localities were genetically disjunct.

## Authors' contributions

C.I.F., J.M.W., M.T. and H.G.S. conceived the ideas; C.I.F. obtained and analysed the data; C.I.F. led the writing; and J.M.W., M.T. and H.G.S assisted with the writing. All authors read and approved the final manuscript.
